# Histological demonstration of BSEP/ABCB11 inhibition in transient neonatal cholestasis: a case report

**DOI:** 10.1186/s12887-020-02201-x

**Published:** 2020-07-09

**Authors:** Anna Baghdasaryan, Lisa Ofner-Ziegenfuß, Carolin Lackner, Peter Fickert, Bernhard Resch, Nicholas Mark Morris, Andrea Deutschmann

**Affiliations:** 1 Division of General Pediatrics, Department of Pediatrics and Adolescent Medicine, Medical University of Graz, Auenbruggerplatz 34/2, 8036 Graz, Austria; 2 Institute for Human Genetics, Medical University of Graz, Graz, Austria; 3 Institute of Pathology, Medical University of Graz, Graz, Austria; 4 Division of Gastroenterology and Hepatology, Department of Internal Medicine, Medical University of Graz, Graz, Austria; 5 Division of Neonatology, Department of Pediatrics and Adolescent Medicine, Medical University of Graz, Graz, Austria

**Keywords:** BSEP deficiency, Transient neonatal cholestasis

## Abstract

**Background:**

Idiopathic or transient neonatal cholestasis (TNC) represents a group of cholestatic disorders with unidentified origin and remains a diagnosis of exclusion. Dysfunction of hepatobiliary transporters mediating excretion of biliary constituents from hepatocytes may play a central role in the pathogenesis of cholestasis. Despite variants of bile salt (BS) export pump (BSEP*/ABCB11*) have already been described in TNC, the pathogenic role of BSEP dysfunction in TNC remained so far elusive.

**Case presentation:**

We report on a newly-identified heterozygous *ABCB11* missense variant (c.1345G > A, p.Glu449Lys) which was associated with prolonged cholestasis in a term infant after a complicated neonatal period. Moreover, we show for the first time almost completely abolished BSEP expression on the hepatocellular membrane in TNC.

**Conclusion:**

This report demonstrates for the first time a close association between the prolonged cholestasis in infancy and impaired BSEP expression on the hepatocyte canalicular membrane in a heterozygous carrier of newly-identified *ABCB11* variant.

## Background

Idiopathic or transient neonatal cholestasis (TNC) represents a group of intrahepatic cholestatic disorders in infancy with poorly understood pathogenesis. It is characterized by onset of cholestasis in the first week of life, elevation of biochemical parameters of liver injury and cholestasis for several months and their normalization usually by the age of 1 year [1]. Interestingly, despite the complicated neonatal period (asphyxia, sepsis, parenteral nutrition, etc.) is associated with a higher incidence of TNC [1], only some of the infants admitted to the neonatal intensive care unit develop TNC, suggesting involvement of genetic predisposing mechanisms.

Bile salt (BS) excretion from hepatocytes is mediated by BS-specific canalicular transporter the bile salt export pump (BSEP/*ABCB11*). Mutations leading to complete loss of BSEP function cause progressive familial intrahepatic cholestasis type 2 (PFIC 2), a severe and progressive cholestatic liver disease, which may lead to development of hepatocellular carcinoma and need for liver transplantation [2]. In contrast, common *ABCB11* variants as well as its inhibition have been associated with transient cases of liver injury such as intrahepatic cholestasis of pregnancy, drug-induced liver injury (DILI), sepsis- and parenteral nutrition (PN)-induced cholestasis [3]. Despite *ABCB11* variants have been associated with TNC [4, 5], the proof for impaired BSEP function in the pathogenesis of TNC has been missing. In particular, no evidence has been shown for persisting BSEP dysfunction after discontinuation of triggering factors. Hence, here we describe a novel heterozygous *ABCB11* variant and provide the histological correlate as a potential molecular mechanism for TNC.

## Case presentation

**Fig. 1:**
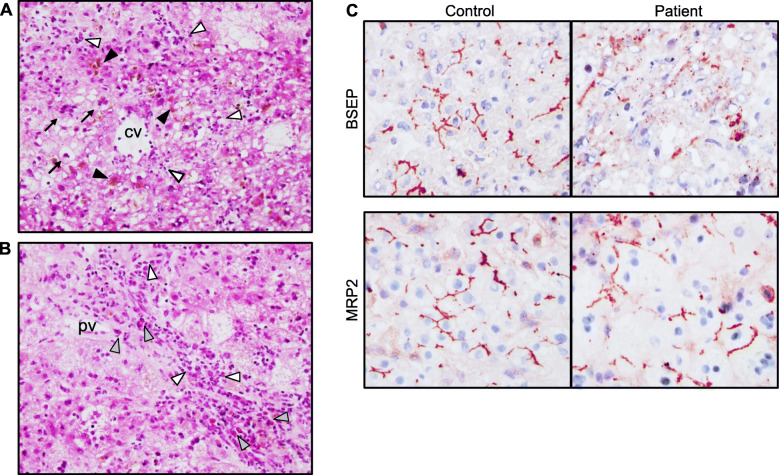
**a.** and **b.** Hepatocellular edema, multinucleated hepatocytes (arrows), accumulation of bile pigment inside the bile canaliculi and hepatocytes (black arrowheads) and mild portal infiltration of neutrophil and eosinophil granulocytes (white and grey arrowheads respectively) is presented. Original magnification: 60 x. pv: portal vein, cv: central vein. **c.** Immunohistochemical stainings for BSEP (BS transporter) and MRP2 (canalicular bilirubin and glutathione transporter) are shown in the control and patient’s liver specimens. As shown, BSEP-positive inclusions retained in the hepatocellular cytoplasm and no transporter was detected on the canalicular membrane. The internal control MPR2 shows preserved canalicular expression. Original magnification 100x. The specificity of immunohistochemical staining was confirmed by negative control (not shown)

A term male newborn was delivered by emergency cesarean section to a 27-year-old primigravida with gestational diabetes. The mother had followed nutritional recommendations and had taken no medication during pregnancy. The newborn was large-for-gestational-age, had an umbilical arterial pH of 6.92 and an APGAR score of 1 at 1 min. After cardiorespiratory resuscitation, his APGAR scores were 8 and 10 at 5 and 10 min respectively. His blood tests revealed acidosis, hypoglycemia, severe multiorgan involvement and impaired coagulation, requiring whole body cooling, mechanical ventilation, empiric antibiotic treatment, analgesia, sedation, PN, glucose infusions and clotting factor replacement in the first week of life. Subsequently, the newborn’s clinical condition and laboratory findings improved, the whole body cooling and mechanical ventilation were stopped on day 4 and 7 respectively and full oral feeding was reached on day 14. Despite laboratory findings of multiorgan involvement returned to normal, ALT elevation and direct hyperbilirubinemia persisted beyond the age of 3 weeks (Table 1). Biliary atresia, congenital infections, α1-antitrypsin deficiency, thyroid dysfunction, cystic fibrosis and metabolic disorders were excluded. Genetic analysis of the main hepatobiliary transporters discovered a novel heterozygous missense variant in the *ABCB11* gene (c.1345G > A, p.Glu449Lys). According to current ACMG guidelines the variant was classified as likely pathogenic [6]. Furthermore, 2 heterozygous variants, both classified as variants of unknown significance were identified in the gene *ATP8B1* encoding the biliary aminophospholipid transporter (c.636 T > A, p.Ile212Ile and c.1819 + 49 T > C). Treatment with ursodeoxycholic acid (UDCA) was initiated on day 28. Due to persisting cholestasis, occasional increase of gamma-glutamyl transferase (GGT) and rising levels of liver transaminases (without an obvious reason) a diagnostic liver biopsy was performed at the age of 3.5 months. Liver histology showed giant cell transformation, lobular inflammation, bile retention inside the hepatocytes and bile canaliculi and mild portal fibrosis and ductal proliferation (Fig. 1a, b). These findings resembled histological presentation of PFIC2 [7]. Specifically, identification of bile plugs within the hepatocytes and bile canaliculi suggested dysfunction of bile elimination mechanisms. Interestingly, immunohistochemical staining revealed almost completely abolished canalicular BSEP expression (Fig. 1c). No additional medication was initiated in our patient. His clinical course improved continuously. UDCA therapy was discontinued at the age of 6 months due to normalization of serum bilirubin levels. Serum liver enzymes returned to normal by the age of 14 months. Our patient showed normal physical development and had normal liver ultrasound findings as well as biochemical liver function tests during the follow-up controls performed on the regular basis till the age of 5 years. Since surrogate parameters of hepatocellular injury and cholestasis improved, we believe that BSEP loss was of transient nature and found it unethical to perform a second liver biopsy for the histochemical proof of recovery.

**Table 1:** Evolution of biochemical parameters of liver injury

Laboratory test	0–24 h	24–72 h	5 day	3 weeks	1.5 months	3 months	6 months	12 months	16 months	45 months
ALT (U/L)	857 (< 67)	485 (< 67)	180 (< 67)	74 (< 67)	268 (< 59)	406 (< 59)	250 (< 45)	71 (< 45)	30 (< 45)	19 (< 54)
AST (U/L)	2145 (< 77)	697 (< 77)	98 (< 77)	89 (< 77)	261 (< 57)	304 (< 57)	112 (< 43)	59 (< 43)	30 (< 43)	28 (< 43)
LDH (U/L)	9082 (< 700)	4628 (< 700)	1583 (< 700)	391 (< 700)	301 (160–430)	306 (160–430)	n.m.	n.m.	257 (120–340)	257 (120–340)
Bilirubin total (mg/dL)	2.5 (< 1.5)	4.8 (< 1.5)	3.38 (< 1.5)	8.08 (< 1.5)	11.07 (0.1–1.2)	5.8 (0.1–1.2)	0.46 (0.1–1.2)	n.m.	0.17 (0.1–1.2)	0.35 (0.1–1.2)
Bilirubin conj. (mg/dL)	n.m.	n.m.	n.m.	n.m.	8.87 (< 0.2)	n.m.	n.m.	n.m.	n.m.	n.m.
AP (U/L)	108 (< 310)	103 (< 310)	n.m.	304 (< 310)	363 (< 380)	n.m.	n.m.	n.m.	127 (< 320)	130 (155–370)
GGT (U/L)	166 (< 216)	165 (< 216)	54 (< 216)	128 (< 216)	83 (< 162)	188 (< 162)	241 (< 76)	52 (< 76)	20 (< 38)	13 (< 38)
Lactate (mmol/L)	13.8 (0.5–2.7)	6.0 (0.5–2.7)	1.4 (0.5–2.7)	0.9 (0.5–2.7)	0.5 (0.5–2.7)	n.m.	n.m.	n.m.	n.m.	n.m.
PT (%)	18 (70–120)	34 (70–120)	78 (70–120)	82 (70–120)	n.m.	98 (70–120)	98 (70–120)	n.m.	n.m.	n.m.

For each parameter, the age-dependent reference ranges are presented in brackets

*ALT* alanine aminotransferase, *AST* aspartate aminotransferase, *AP* alkaline phosphatase, *GGT* gamma-glutamyl transferase, *LDH* lactate dehydrogenase, *n.m*. not measured, *PT* prothrombin time

## Discussion and conclusions

We report on a previously unknown heterozygous *ABCB11* variant and provide for the first time the histological proof of BSEP decompensation in TNC after severe neonatal asphyxia. Ischemic liver injury occurs as part of so-called “multi-organ damage” following perinatal asphyxia and becomes manifest biochemically in peaks of LDH and liver enzymes within the first 24–72 h. These elevated parameters usually decrease in the following days and return to normal between 6 and 12 days after the hypoxic event [8]. However, prolonged cholestasis in our patient was inconsistent with a course of asphyxia-related liver injury (Table 1). Moreover, evolution of serum liver tests suggested that in contrast to asphyxia-mediated massive hepatocellular damage immediately after birth, impairment of bile elimination mechanisms were responsible for chronic liver injury (ALT/AP ratio of 1.4 at the age of 3 months vs. ALT/AP ratio of 8 on day 1 *post-partum*). Genetic analysis determined a new heterozygous variant of *ABCB11*, a unique BS-specific transporter, which loss cannot be compensated by an alternative export mechanism in humans. Due to limited size of the liver specimen and based on data obtained from the genetic analysis, examination of potential molecular mechanisms causing cholestasis was restricted to studying BSEP as the most likely candidate. Although the identified mutation was found in a heterozygous state and GGT levels did not match with classical BSEP deficiency, histologically BSEP was almost abolished from hepatocyte canalicular membrane in our patient. Since clinical severity of different *ABCB11* mutations directly correlates with the amount of mature protein on hepatocellular membrane [2, 3], impaired canalicular BSEP expression is likely to play at least a partial role in the pathogenesis of prolonged cholestasis in our patient. Of note, the mother of our patient carries the same mutation with no cholestatic events in her history, indicating that the heterozygous state of the identified mutation is likely to be sufficient to maintain BS excretion under normal conditions. In addition, occasionally elevated GGT levels in our patient were inconsistent with classical presentation of BSEP deficiency in PFIC2. It can, therefore, be speculated that the complicated neonatal period and multifactorial liver injury played a critical role in transient decompensation of *ABCB11* originating from non-mutated gene copy. It still remains unclear which mechanisms are responsible for long-term BSEP inhibition in our patient. BSEP is believed to recycle between the plasma membrane and subapical vesicles and its half-life in the apical membrane ranges from 4 to 6 days [3]. This may explain short-term BSEP decompensation and its rapid recovery after triggering factor has resolved, as reported in cases of hypoxia-, sepsis-, PN-induced cholestasis or DILI. In contrast, mechanisms responsible for persisting BSEP dysfunction long after termination of damaging factors, as observed in our patient, remain so far elusive. Interestingly, prolonged cholestasis has been observed in rare cases of DILI [9]. However, the pathogenesis of DILI is complex and involves -in addition to BSEP inhibition- additional mechanisms [10, 11]. This is supported by the fact that only a very low percent of newborns with drug intake during the complicated neonatal course develop TNC. In this context, indirect inhibition of *BSEP* by other hepatobiliary transporters through the farnesoid X receptor (FXR)-mediated signaling has to be considered. Despite two heterozygous mutations found in *ATP8B1* were of unknown significance, BSEP inhibition through *ATP8B1-FXR*-mediated mechanism cannot be excluded [12] and ATP8B1 dysfunction should be considered as additional potentially pathogenic mechanism in course of transient cholestasis in our patient. We believe that prolonged BSEP deficiency and resulting intracellular cholestasis were of multifactorial origin in our patient. In this context, persisting hepatocyte inflammatory response triggered by initial massive liver and multiorgan damage and maintained by ongoing interplay of drugs/toxins, mitochondrial inflammatory reaction by accumulating bile acids [13, 14] as well as immaturity of regulatory mechanisms are likely to contribute to BSEP inhibition. However, BSEP inhibition itself, even if secondary to inflammation or toxicity by endogenous intermediates, played important role for the maintenance of prolonged cholestasis in our patient.

In this report, we describe a novel heterozygous *ABCB11* variant and provide, for the first time, the histological demonstration of canalicular BSEP deficiency in a patient with complicated neonatal period and TNC. The role of the newly-identified mutation as a potentially predisposing factor for development of cholestasis has to be investigated in future research.

## Data Availability

All data generated or analyzed during this study are included in this published article.

## Abbreviations

ALT: alanine aminotransferase,
AP: alkaline phosphatase,
AST: aspartate aminotransferase,
BS: bile salt,
BSEP: bile salt export pump,
cv: central vein,
DILI: drug-induced liver injury,
FXR: farnesoid X receptor,
GGT: gamma-glutamyl transferase,
LDH: lactate dehydrogenase,
n.m.: not measured,
PFIC 2: progressive familial intrahepatic cholestasis type 2,
PN: parenteral nutrition,
PT: prothrombin time,
pv: portal vein,
TNC: transient neonatal cholestasis,
UDCA: ursodeoxycholic acid
